# Frequency and pattern of Chinese herbal medicine prescriptions for urticaria in Taiwan during 2009: analysis of the national health insurance database

**DOI:** 10.1186/1472-6882-13-209

**Published:** 2013-08-15

**Authors:** Pei-Shan Chien, Yu-Fang Tseng, Yao-Chin Hsu, Yu-Kai Lai, Shih-Feng Weng

**Affiliations:** 1 Department of Chinese Medicine, Chi Mei Medical Center, Liouying, Taiwan; 2 Department of Chinese Medicine, Chi Mei Medical Center, Yongkang, Taiwan; 3 Department of Medical Research, Chi Mei Medical Center, Yongkang, Taiwan; 4 Department of Hospital and Health Care Administration, Chia Nan University of Pharmacy and Science, Tainan, Taiwan

**Keywords:** Urticaria, Chinese herbal medicine, National health insurance database, Taiwan

## Abstract

**Background:**

Large-scale pharmaco-epidemiological studies of Chinese herbal medicine (CHM) for treatment of urticaria are few, even though clinical trials showed some CHM are effective. The purpose of this study was to explore the frequencies and patterns of CHM prescriptions for urticaria by analysing the population-based CHM database in Taiwan.

**Methods:**

This study was linked to and processed through the complete traditional CHM database of the National Health Insurance Research Database in Taiwan during 2009. We calculated the frequencies and patterns of CHM prescriptions used for treatment of urticaria, of which the diagnosis was defined as the single ICD-9 Code of 708. Frequent itemset mining, as applied to data mining, was used to analyse co-prescription of CHM for patients with urticaria.

**Results:**

There were 37,386 subjects who visited traditional Chinese Medicine clinics for urticaria in Taiwan during 2009 and received a total of 95,765 CHM prescriptions. Subjects between 18 and 35 years of age comprised the largest number of those treated (32.76%). In addition, women used CHM for urticaria more frequently than men (female:male = 1.94:1). There was an average of 5.54 items prescribed in the form of either individual Chinese herbs or a formula in a single CHM prescription for urticaria. Bai-Xian-Pi (*Dictamnus dasycarpus* Turcz) was the most commonly prescribed single Chinese herb while Xiao-Feng San was the most commonly prescribed Chinese herbal formula. The most commonly prescribed CHM drug combination was Xiao-Feng San plus Bai-Xian-Pi while the most commonly prescribed triple drug combination was Xiao-Feng San, Bai-Xian-Pi, and Di-Fu Zi (*Kochia scoparia*).

**Conclusions:**

In view of the popularity of CHM such as Xiao-Feng San prescribed for the wind-heat pattern of urticaria in this study, a large-scale, randomized clinical trial is warranted to research their efficacy and safety.

## Background

Urticaria is defined as a kind of skin rash notable for pale red, itchy bumps caused by allergic reactions to internal and external agents. The word ‘urticaria’ is derived from the Latin word urtica, which means ‘nettle’, which is a tooth-leaved plant covered with hairs that secret a stinging fluid that immediately affects the skin [1]. According to a previous study, more than 50% of patients continue to tolerate chronic urticaria 10 years after their initial diagnosis, and most of them need long-term treatment [2].

Urticaria is a disorder affecting up to 25% of people in the United States (US). Chronic urticaria is defined as repeated episodes of symptoms that last for more than 6 weeks. The majority of cases (> 80%) have an unknown cause, which is called chronic idiopathic urticaria (CIU). The incidence of CIU is higher in women than in men (2:1), but not among atopic patients, with an evaluated prevalence of up to 1% in the US. CIU patients report quality of life impairment similar to patients who have cardiac disease and other chronic skin diseases such as atopic dermatitis and psoriasis. In addition, these patients who did not use immunosuppressants and who attended a university clinic had annual health care costs of more than US $2,000 [3].

Urticaria is caused by an allergic response to an allergenic substance. Drugs commonly used for treating urticaria include antihistamines, omalizumab, cyclosporine, and low-dose corticosteroids [4]. Side effects from antihistamines are likely to occur, especially in the elderly population. Antihistamines have potent anticholinergic properties, which decrease urinary flow and can lead to urinary retention. Other side effects such as confusion, dizziness, drowsiness, fatigue, dryness or CNS-altering effects are also more likely to occur in elderly patients [5]. Corticosteroids are not recommended for sustained use because of the risk of weight gain, hypertension, osteoporosis, and cataracts [4].

Throughout the world, many people use complementary and alternative medicine, and Traditional Chinese Medicine (TCM) is one of the most popular forms. In Western populations, the reasons for the use of TCM include experiencing failure of standard health care, the need for autonomy, and preference for natural therapies [6-8]. Based on the 2007 National Health Interview Survey, the prevalence of complementary and alternative medicine use was 38% in American adults [9]. Unlike the health system in many Western countries, TCM is regarded as an important part of the Chinese health care system. In Chinese populations, the deep trust in the efficacy of TCM stems from people’s faith in cultural wisdom and heritage. The reasons for the use of TCM include searching for tonic care or health promotion, individualizing treatment to suit different health needs, causing few side effects, and delivering therapeutic effects that “clear the root of the disease” [10]. Based on a large-scale survey in China in 2009, the prevalence of TCM use was 19.2%, which translates into 0.67 billion visits/year [11]. According to a large-scale, cross-sectional study of TCM utilization in Hong Kong in 2002, from among patients who claimed to have medical benefits or insurance policies, 14.5% were covered for TCM [12].

The National Health Insurance (NHI) program was initiated in Taiwan in 1995, and TCM has been covered by the NHI since 1996. Currently, the NHI covers about 99% of the 23 million population of Taiwan [13]. Citizens in Taiwan are free to choose Western medicine or TCM. According to the results of a large-scale investigation of the use of TCM in Taiwan from 1996 to 2001, there was a steady increase in the annual number of TCM users, and 62.5% of people used TCM covered by the NHI during this period [14].

Because TCM is inexpensive and widely available, it has been used for the treatment of skin diseases for centuries. Additionally, some controlled clinical studies showed that TCM was effective and safe for treatment of inflammatory skin disorders [15]. Nonetheless, there are no large scale pharmaco-epidemiologic studies of CHM for the treatment of urticaria. The aim of this study was to explore the frequencies and patterns of CHM prescriptions for urticaria by analysing the population-based CHM database in Taiwan.

## Methods

### Data sources

Taiwan began the National Health Insurance program in 1995 [16]. The National Health Research Institutes transferred national health insurance reimbursement data into files for research. These files provided detailed health care services information for each patient, including all payments for outpatient visits, hospitalizations, and prescriptions. For each outpatient visit or hospitalization, the data contained up to 3–5 diagnoses coded under the *International Classification of Diseases, Ninth Revision*, along with the prescription drugs and doses, special treatments, and dates of these orders.

In Taiwan, all TCMs are provided only in ambulatory clinics. In addition, only licensed TCM doctors are qualified for reimbursement [17]. In this study, we used the complete TCM claims database for 2009 from the NHI Research Database (NHIRD) released by the National Health Research Institute in Taiwan, including the details of ambulatory care prescriptions in every corresponding TCM claim (CM_CD2009.dat and CM_OO2009.dat); no inpatient care included TCM. Because the identification numbers of all individuals in the NHIRD were encrypted to protect patient privacy, this study was exempt from a full review by the institutional review board.

### Study design

In Taiwan, TCM doctors are asked to make diagnoses based on ICD-9-CM coding. In this study, we used data from patients with the single diagnostic code for urticaria (i.e., ICD-9 code 708). From all 38,547,753 visits in the CM_CD2009.dat database, 37,386 subjects visited TCM clinics for urticaria in Taiwan during 2009. Among these subjects, a total of 95,765 prescriptions with CHM were recorded. The drug list for CHM was obtained from the Bureau of Health Promotion, and the database (CM_CD2009 and CM_OO2009) was interlinked with six variables (year and month of the fee, type of application, hospital code to identify each hospital, date of application, type of case, and serial number) in the database.

According to the TCM theory, a single prescription from a TCM doctor may contain four ingredients: 1) a single Chinese herb, 2) Fu-Fang, 3) Fang-Ji (regimen or remedy) or 4) Chia-Chien-Fang. Fu-Fang is composed of multiple herbs of various dosages. Fang-Ji is a combination of compatible Chinese herbs in fixed dosages according to classical or well-known Chinese textbooks of medicine. Chia-Chien-Fang is a classical formula in which a drug ingredient is combined with Chinese herbs. These ingredients are made of powder and can be easily mixed in a single prescription [16].

### Statistical analysis

Mining frequent itemsets and rules of association are popular and well researched methods for discovering interesting relations between variables in large databases. Piatetsky-Shaprio (1991) first described strong rules for database mining and analysis using measures of interestingness and association [18].These itemset rules have been applied to many diverse disciplines, such as marketing [19] and computer science [20,21]. In the past decade, they have also been introduced into the medical area [22-24].

In this study, a data mining process was used to help discover and characterise prescription patterns of the Chinese herbal drugs or formulae for urticaria. The frequent itemset mining method was applied to evaluate co-prescriptions of CHM. The support (%) of a prescribed drug set X was defined as the proportion of all prescriptions in the data set that contained the drug set X. Support is a measure of how frequently the rule occurs in the database. The Statistical Software R (version 2.13.2) package ‘arules’ was used, and the function ‘apriori’ within a minimum support of 1% was set to perform the analyses.

## Results

### Patient features

There were 37,386 subjects who visited TCM clinics for urticaria in Taiwan during 2009. Among these subjects, a total of 95,765 prescriptions for CHM were recorded. Almost two thirds of the patients treated for urticaria with CHM were between 18 and 50 years of age (Table 1). Female subjects used CHM for urticaria almost twice as frequently as male subjects (female:male = 1.94:1).

**Table 1:** Age-specific frequencies for the use of Chinese herbal medicines among patients with urticaria

**Age (years)**	**Subjects with urticaria using Chinese herbal medicines**		
	**No. of patients (%)**	**Males (%)**	**Females (%)**
0–18	6001 (16.05)	2912 (7.79)	3089 (8.26)
18–35	12246 (32.76)	3546 (9.49)	8700 (23.27)
35–50	11889 (31.80)	3464 (9.26)	8425 (22.54)
50–65	5572 (14.90)	2045 (5.47)	3527 (9.43)
> 65	1678 (4.49)	734 (1.96)	944 (2.53)
Total	37386 (100%)	12701 (33.97)	24685 (66.03)

Male:female = 1:1.94.

### Chinese herbal formulae

Xiao-Feng San (48.84%) was by far the most commonly prescribed Chinese herbal formula for subjects with urticaria. The remaining half of prescriptions comprised 10 other CHM formulations (Table 2).

**Table 2:** Top 10 Chinese herbal formulae prescribed for urticaria in Taiwan during 2009

**Chinese herbal formula**	**Ingredients**	**No. of prescriptions**	**Percentage**
Xiao Feng San	*Angelicae sinensis, Rehmannia glutinosa* Liboschitz, *Saposhinkoviae* Radix, *Anemarrhena* Rhizome, *Sophora Flavescens*, *Sesamum Indicum*, *Schizonepeta tenuifolia*, *Atractylodes* Rhizome, *Arctium lappa*, *Gypsum* Fibrosum, *Glycyrrhiza uralensis*, *Cryptotympana atrata* Fabr, *Akebia quinata* Decne	46772	48.84%
Jing Fang Bai Du San	*Bupleurum falcatum*, *Ligusticum chuanxiong* Hort, *Saposhinkoviae* Radix, *Poriae cocos*, *Platycodon grandiflorum*, *Schizonepeta tenuifolia*, *Notopterygii* Rhizoma, *Glycyrrhiza uralensis*, *Angelicae Tuhou* Radix, *Poncirus trifoliata* Rafin, *Zingiber officinale*	14561	15.20%
Huang-Lian-Jie-Du-Tang	*Coptidis* Rhizoma, *Gardenia jasminoides*, *Scutellaria baicalensis*, *Phellodendri* cortex	8837	9.23%
Dang Gui Yin Zi	*Angelicae sinensis*, *Rehmannia glutinosa* Liboschitz, *Paeoniae lactiflorae* Radix, *Ligusticum chuanxiong* Hort, *Polygonum multiflorum*, *Schizonepeta tenuifolia*, *Saposhinkoviae* Radix, *Tribulus terrestris*, *Astragalus membranaceus*, *Glycyrrhiza uralensis*, *Zingiber officinale*	7575	7.91%
Longdan Xiegan Tang	*Gardenia jasminoides*, *Alismatis* Rhizoma, *Gentiana scabra*, *Akebia quinata* Decne, *Rehmannia glutinosa* Liboschitz, *Bupleurum falcatum*, *Glycyrrhiza uralensis*, *Plantaginis semen*, *Angelicae sinensis*	7554	7.89%
Jia Wei Xiao Yao San	*Bupleurum falcatum*, *Angelicae sinensis*, *Paeoniae lactiflorae* Radix, *Atractylodes macrocephala*, *Poriae cocos*, *Glycyrrhiza uralensis*, *Paeonia suffruticosa* Andr, *Gardenia jasminoides*, *Menthae folium*, *Zingiber officinale*	7007	7.32%
Wen Ching Yin	*Gardenia jasminoides*, *Scutellaria baicalensis*, *Coptidis* Rhizoma, *Phellodendri* cortex, *Ligusticum chuanxiong* Hort, *Rehmannia glutinosa* Liboschitz, *Angelicae sinensis, Paeoniae lactiflorae* Radix	6908	7.21%
Gui Zhi Tang	*Cinnamomi ramulus, Paeoniae lactiflorae* Radix*, Glycyrrhiza uralensis, Zingiber officinale, Zizyphus jujuba* Mill	4600	4.80%
Yin Qiao San	*Lonicera japonica, Forsythia suspensa, Platycodon grandiflorum, Arctium lappa, Menthae folium, Glycyrrhiza uralensis, Phragmites communis* Trin.*, Lophatherum gracile, Schizonepeta tenuifolia*	3495	3.65%
Xiao Chai Hu Tang	*Bupleurum falcatum, Pinellia ternata, Panax ginseng, Glycyrrhiza uralensis, Scutellaria baicalensis, Zizyphus jujuba* Mill*, Zingiber officinale*	3377	3.53%

Total prescription numbers, n = 95765.

### Single Chinese herbs

The top four most commonly prescribed herbs for urticaria were *Dictamnus dasycarpus* Turcz (Bai-Xian-Pi) (15.55%), *Paeonia suffruticosa* Andr (Mu-Dan-Pi) (13.21%), *Kochia scoparia* (Di-Fu-Zi) (12.75%) and *Forsythia suspensa* (Lian-Qiao) (12.50%). The prescription rates for each of the remaining herbs were less than 10% (Table 3).

**Table 3:** The top 10 individual Chinese herbs prescribed for urticaria in Taiwan during 2009

**Chinese single herb (Chinese name)**	**Botanical name**	**No. of prescriptions**	**Percentage**
Bai-Xian-Pi	*Dictamnus dasycarpus* Turcz	14895	15.55%
Mu Dan Pi	*Paeonia suffruticosa* Andr	12646	13.21%
Di Fu Zi	*Kochia scoparia*	12213	12.75%
Lian-Qiao	*Forsythia suspensa*	11966	12.50%
Chan Tui	*Cryptotympana atrata* Fabr	9408	9.82%
Yi Yi Ren	*Semen coicis*	8982	9.38%
Gan Cao	*Glycyrrhiza uralensis*	8643	9.03%
Jing Jie	*Schizonepeta tenuifolia*	7814	8.16%
Jin Yin Hua	*Lonicera japonica*	7803	8.15%
Tu Fu Ling	*Smilax lanceifolia* Roxb	7743	8.09%

Total prescription number, n = 95765.

### Combinations of CHM

**Figure 1:**
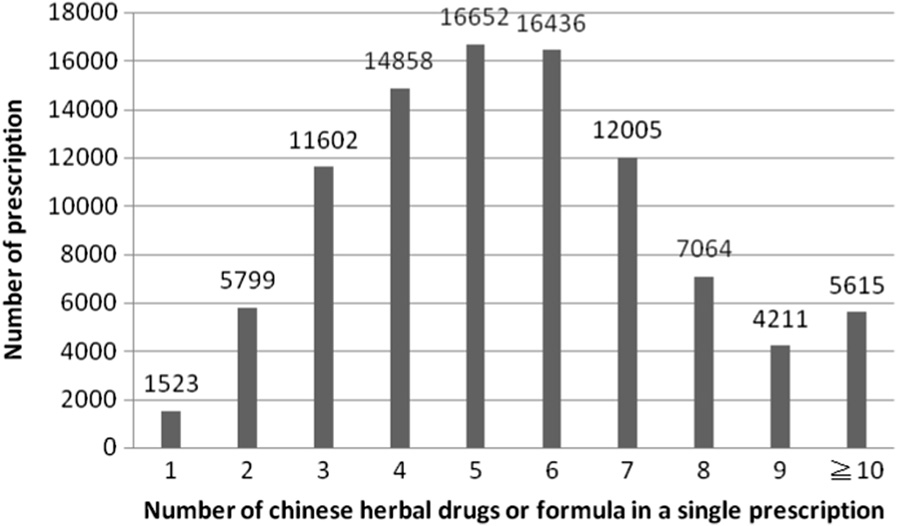
**Relationship between the number of prescriptions and the number of Chinese herbs.** The average is 5.54 ± 2.39 Chinese herbal items in a single prescription for treatment of subjects with urticaria (total prescription number, n = 95765).

On average, a single prescription of CHM for urticaria contained 5.54 different herbs. The most common prescriptions of CHM combinations or single Chinese herbs contained 4 to 6 herbs (Figure 1). Based on frequent itemset mining, the most commonly prescribed pattern of a 2-drug combination of CHM for urticaria treatment was Xiao Feng San plus *Dictamnus dasycarpus* Turcz (Table 4), while the 3-drug combination was Xiao Feng San, *Dictamnus dasycarpus* Turcz and *Kochia scoparia* (Table 5).

**Table 4:** The most common two-drug combinations of Chinese herbal medicines in a single prescription for urticaria

**Chinese herbal formulae or drugs**		**No. of prescriptions**	**Support**
**First**	**Second**		
Xiao Feng San	*Dictamnus dasycarpus* Turcz	8938	9.33%
Xiao Feng San	*Kochia scoparia*	8057	8.41%
Xiao Feng San	Jing Fang Bai Du San	6252	6.53%
Xiao Feng San	*Paeonia suffruticosa* Andr	6044	6.31%
*Kochia scoparia*	*Dictamnus dasycarpus* Turcz	5475	5.72%
Xiao Feng San	*Cryptotympana atrata* Fabr	5037	5.26%
Xiao Feng San	*Smilax lanceifolia* Roxb	4523	4.72%
Xiao Feng San	*Forsythia suspensa*	4386	4.58%
Xiao Feng San	Dang Gui Yin Zi	4325	4.52%
Xiao Feng San	*Semen coicis*	4212	4.40%

Total prescription number, n = 95765.

**Table 5:** The most common three-drug combination of Chinese herbal medicines in a single prescription for urticaria

**Chinese herbal formulae or drug**			**No. of prescriptions**	**Support**
**First**	**Second**	**Third**		
Xiao Feng San	*Dictamnus dasycarpus* Turcz	*Kochia scoparia*	3693	3.86%
Xiao Feng San	*Dictamnus dasycarpus* Turcz	*Paeonia suffruticosa* Andr	1832	1.91%
Xiao Feng San	*Lonicera japonica*	*Forsythia suspensa*	1335	1.39%
Xiao Feng San	*Dictamnus dasycarpus* Turcz	*Smilax lanceifolia* Roxb	1292	1.35%
Xiao Feng San	*Dictamnus dasycarpus* Turcz	*Cryptotympana atrata* Fabr	1287	1.34%
Xiao Feng San	*Kochia scoparia*	*Paeonia suffruticosa* Andr	1281	1.34%
Xiao Feng San	*Kochia scoparia*	Jing Fang Bai Du San	1224	1.28%
Xiao Feng San	*Kochia scoparia*	*Cryptotympana atrata* Fabr	1131	1.18%
Xiao Feng San	*Paeonia suffruticosa* Andr	*Rehmannia glutinosa* Liboschitz	1126	1.18%
Xiao Feng San	*Kochia scoparia*	*Smilax lanceifolia* Roxb	1096	1.14%

Total prescription number, n = 95765.

## Discussion

In this study, Xiao-Feng San (48.84%) was by far the most commonly prescribed Chinese herbal formula for subjects with urticaria. According to the principles of TCM treatment for urticaria, Xiao-Feng San, which expels wind and clears heat, is prescribed for TCM patterns of Wind and Heat. Xiao-Feng San is famous for its antipruritic effect and is frequently used to treat chronic skin diseases such as urticaria [25]. In a rat model study, it revealed that Xiao-Feng San reduced delayed-type hypersensitivity responses by decreasing the level of interleukin-2 [25]. Some ingredients of Xiao-Feng San are reported to have anti-inflammatory actions such as *Saposhnikoviae radix* has significant anti-inflammatory effects [26]. An aqueous extract of *Rehmannia glutinosa* dose-dependently inhibited skin allergic reactions activated by anti-dinitrophenyl (DNP) IgE [27]. Another study reported that *Glycyrrhiza uralensis* had macrophage immunomodulatory activity [28]. On the other hand, another popular Chinese herbal formula used in treating urticaria noted in our study was Huang-Lian-Jie-Du-Tang (9.23%). It is reported to have anti-inflammatory effects such as inhibition of interleukin-8 production, nitric oxide production in macrophages, and inflammation-induced mRNA expression of neuropeptides [29].

With regard to a single Chinese herb, Bai-Xian-Pi (*Dictamnus dasycarpus* Turcz) (15.55%) was the most commonly used to treat urticaria. It is reported to have anti-allergic effects and directly inhibits scratching behaviour and vascular permeability induced by histamine and serotonin release [30]. The second commonly used Chinese herb was Mu Dan Pi (*Paeonia suffruticosa* Andr) (13.21%). According to TCM theory, Mu Dan Pi clears heat from the blood [31]. *P. suffruticosa* Andr contains five compounds including paeonol, paeoniflorin, paeonoside, paeonollide and apiopaeonoside; among them, paeonol is the main bioactive component [32]. It had potent anti-inflammatory and analgesic effects in a rat model of carrageenan-evoked thermal hyperalgesia [32].

Our results showed that Di-Fu-Zi (*Kochia scoparia*) (12.75%) is the third commonly used Chinese herb in treating urticaria. The fruits of *Kochia scoparia* have been used in TCM for centuries to treat skin diseases. Momordin Ic is the main active component in the fruits of *K. scoparia*. Studies indicate that it has a peripheral antinociceptive effect mediated by anti-inflammatory action [33]. *K. scoparia* is a potent inhibitor of lipopolysaccharide-induced nitric oxide, prostaglandin E_2_ and tumour necrosis factor alpha production [34]. Arctiin, isolated from *Forsythiae* fructus, inhibits the effects of lipopolysaccharide by repressing a key inflammatory pathway related to NF-kB, prostaglandin E_2_ and nitric oxide production, and expression of proinflammatory cytokines [35].

Other commonly prescribed Chinese herbs used for treatment of urticaria identified in our study included *Glycyrrhiza uralensis* and *Schizonepeta tenuifolia*, which have strong anti-inflammatory, anti-oxidative stress and detoxification properties [36,37]. Besides, *Lonicera japonica* exhibits anti-inflammatory activity through the inhibition of cyclo-oxygenase-2 (COX-2), inducible nitric oxide synthase (iNOS), and cytokines such as TNF-α, interleukin-1β and interleukin-6 by inhibiting the p38 mitogen-activated protein kinases and NF-κB pathways [[38]. These Chinese herbs are also worthy of further investigations of their clinical efficacy and safety for treating urticaria.

In this study, we explored the frequencies and patterns of CHM prescriptions for urticaria by analysing the population-based CHM database in Taiwan during 2009. Various CHM prescriptions are used to treat urticaria according to physicians’ personal experiences or based on traditional Chinese texts. However, it remains unclear which of the CHM prescriptions are the most effective in treating urticaria in clinical practice. We observed drug utilization and prescription patterns through a large scale survey of clinical practices, which served as an effective tool for investigating the clinical pharmacology of these compounds. In addition, this study provides relevant information to discern potentially effective CHM for treating urticaria. According to the principles of TCM diagnosis, syndrome differentiation and treatment, we could also summarize TCM patterns and explore the core patterns of urticaria from the results of this study. For example, if the symptoms include red wheals, aversion to wind, thirst, restlessness, a red tongue and rapid pulse, they would be subclassified as the Wind Heat pattern for which a formula such as Xiao-Feng San could be prescribed. On the other hand, if the symptoms include dry skin, pale face and lips, dizziness, a pale tongue and thin pulse, they would be subclassified as the Blood Deficiency pattern. Therefore, a formula such as Dang Gui Yin Zi would be administered. On the basis of the results of this study, future studies could concentrate on the most common TCM patterns found for urticaria, and different treatments could be designated for specific TCM patterns.

Our study has two limitations. First, syndrome differentiation (bianzheng lunzhi) in TCM is the comprehensive analysis of clinical information gained by the four main diagnostic methods: inspection, auscultation or olfaction, inquiry, and palpation. They were written in the medical records by the treating physicians. The exact syndrome differentiation helps physicians select the right herbal formula to provide the best treatment for patients in certain phases of a disease. However, the identities of the patients were encrypted in the NHI reimbursement database; we could not obtain the medical records of the patients. Thus, we could not analyse the exact syndrome differentiation. Second, TCM patients might have received prior Western medicine treatment or be under concomitant therapies, and the therapeutic effect of Chinese medicine could be overestimated.

## Conclusions

In conclusion, we investigated health care claims data in a population-based pharmaco-epidemiology study of Chinese herbs for treating urticaria. The most commonly prescribed Chinese herbal formula for the treatment of urticaria is Xiao-Feng San, and the top three individual herbs are Bai-Xian-Pi, Mu Dan Pi and Di Fu Zi. The therapeutic effects and safety of these commonly used Chinese herbal formulae or individual herbs require further examinations through clinical studies or well-designed randomized, double blind, placebo-controlled trials.

## Competing interests

The authors declare that they have no competing interests.

## Authors’ contributions

All authors were involved in the design and writing of the study, and SFW conducted the statistical analysis. All authors approved the submitted version of the manuscript.

## Pre-publication history

The pre-publication history for this paper can be accessed here:

http://www.biomedcentral.com/1472-6882/13/209/prepub
